# Inflammatory Bowel Disease and COVID-19: How Microbiomics and Metabolomics Depict Two Sides of the Same Coin

**DOI:** 10.3389/fmicb.2022.856165

**Published:** 2022-03-21

**Authors:** Gian Mario Cortes, Maria Antonietta Marcialis, Flaminia Bardanzellu, Angelica Corrias, Vassilios Fanos, Michele Mussap

**Affiliations:** ^1^Neonatal Intensive Care Unit, Department of Surgical Sciences, University of Cagliari, Monserrato, Italy; ^2^Laboratory Medicine, Department of Surgical Sciences, School of Medicine, University of Cagliari, Monserrato, Italy

**Keywords:** inflammatory bowel disease, Crohn’s disease, ulcerative colitis, SARS-CoV-2, COVID-19, metabolomics, microbiomics

## Abstract

The integrity of the gastrointestinal tract structure and function is seriously compromised by two pathological conditions sharing, at least in part, several pathogenetic mechanisms: inflammatory bowel diseases (IBD) and coronavirus disease 2019 (COVID-19), caused by the severe acute respiratory syndrome coronavirus 2 (SARS-CoV-2) infection. IBD and COVID-19 are marked by gut inflammation, intestinal barrier breakdown, resulting in mucosal hyperpermeability, gut bacterial overgrowth, and dysbiosis together with perturbations in microbial and human metabolic pathways originating changes in the blood and fecal metabolome. This review compared the most relevant metabolic and microbial alterations reported from the literature in patients with IBD with those in patients with COVID-19. In both diseases, gut dysbiosis is marked by the prevalence of pro-inflammatory bacterial species and the shortfall of anti-inflammatory species; most studies reported the decrease in *Firmicutes*, with a specific decrease in obligately anaerobic producers short-chain fatty acids (SCFAs), such as *Faecalibacterium prausnitzii*. In addition, *Escherichia coli* overgrowth has been observed in IBD and COVID-19, while *Akkermansia muciniphila* is depleted in IBD and overexpressed in COVID-19. In patients with COVID-19, gut dysbiosis continues after the clearance of the viral RNA from the upper respiratory tract and the resolution of clinical symptoms. Finally, we presented and discussed the impact of gut dysbiosis, inflammation, oxidative stress, and increased energy demand on metabolic pathways involving key metabolites, such as tryptophan, phenylalanine, histidine, glutamine, succinate, citrate, and lipids.

## Introduction

Since the onset of the pandemic outbreak caused by the severe acute respiratory syndrome coronavirus 2 (SARS-CoV-2), it emerged that frailty, elderly, and pre-existing chronic diseases, such as chronic kidney disease, hypertension, cardiovascular disease, and diabetes, are risk factors for the development of severe and/or fatal coronavirus disease 2019 (COVID-19; Grasselli et al., 2020; Falandry et al., 2021). Theoretically, patients with immune-mediated inflammatory diseases, such as inflammatory bowel disease (IBD), might be at increased risk of developing severe COVID-19. However, current knowledge on the pathophysiology of IBD and COVID-19 points out that patients with IBD are not at increased risk or have adverse outcomes for COVID-19 (Neurath, 2020). Strong evidence supporting this conclusion emerge from clinical studies published elsewhere (Allocca et al., 2020; D'Amico et al., 2020), including the discovery that biological therapies (e.g., monoclonal antibodies) may play a protective role against the cytokine storm observed in the course of the SARS-CoV-2 infection (Allocca and Craviotto, 2021). IBD and COVID-19 may share many alterations in molecular mechanisms, microbial communities, and biochemical pathways; “omics” technologies may considerably contribute to decipher mechanisms inducing these alterations, improving patient care and outcome. Microbiome and metabolome were primarily investigated in IBD, and similar but relatively few studies were conducted in patients with COVID-19; in this review, we examined analogies and differences in gut microbiota and body fluids metabolome between IBD and COVID-19 with the aim to identify microbial and metabolic hallmarks linking IBD, COVID-19 and SARS-CoV-2 infection.

## SARS-CoV-2 Infection in Patients With IBD

IBD is an umbrella term encompassing a group of disorders, namely, Crohn’s disease (CD), ulcerative colitis (UC), and inflammatory bowel disease, type unclassified (IBDU; Satsangi et al., 2006). IBD is marked by chronic relapsing–remitting or continuously active idiopathic inflammation and bowel injuries; both adults and children exhibit an immunological dysregulation. The etiology of IBD is multifactorial, including the contribution of genetic, environmental, host factors and their reciprocal interactions (Flynn and Eisenstein, 2019); recent data indicate a worldwide 0.3% incidence and prevalence of IBD (Ng et al., 2017). The therapeutic treatment of IBD with immunomodulators (Park et al., 2020) and biologics (Neurath, 2019) may activate a transient or persistent immunocompromised state inducing opportunistic infections, especially when multiple drugs are prescribed simultaneously (Bonovas et al., 2016; Shah et al., 2017; Irving et al., 2021). Several research groups investigated whether patients with IBD may be or not more susceptible to developing SARS-CoV-2 infection (Monteleone and Ardizzone, 2020), how they should be managed in the context of the COVID-19 pandemic, and the risks and benefits of the therapeutic treatment with immunomodulators, especially in the pediatric age (Dipasquale et al., 2020; Sultan et al., 2020). An early analysis of data collected from the international registry Surveillance Epidemiology of Coronavirus Under Research Exclusion for Inflammatory Bowel Disease (SECURE-IBD) showed that among 525 pediatric and adult patients with IBD and confirmed COVID-19, 31% were hospitalized, 7% developed severe COVID-19, and 3% died (Brenner et al., 2021). Data from the registry evidenced that among patients with IBD, corticosteroids treatment may be a key risk factor for severe COVID-19 (Brenner et al., 2020), confirming results previously reported elsewhere (Mazza et al., 2020). A recent multicenter study enrolling 1816 patients with IBD treated with biologic therapy over the first 2 months of the pandemic reported an overall COVID-19 incidence of 3.9 per 1,000 patients with a 57% hospitalization rate and 29% case fatality rate (CFR; Ardizzone et al., 2021). In a cohort of 1912 patients with an IBD median duration of 17 years, the crude incidence rate of COVID-19 was at 6.2 cases per 1,000 patients, lower than that found in the general population (6.6 cases per 1,000 individuals); the mortality rate was 0.9 per 1,000 and 1 per 1,000 in patients with IBD and the general population, respectively (Taxonera et al., 2020). Although the CFR for IBD cases with COVID-19 was higher than in the general population (16.7 vs. 13.2%, respectively), the statistical difference was not significant. Finally, a meta-analysis including 9,177 patients with IBD from eight studies reported an incidence of 0.3% for COVID-19; 8.6% required admission to the intensive care unit, and the mortality rate was 6.3% (Aziz et al., 2020). In a cohort of patients with IBD, the rate of positive results for anti-SARS-CoV-2 antibodies (approximately 4.6% for IgG and IgM, and 6% for IgA) was found higher than that in healthcare professionals without inflammatory diseases (approximately 1.6% for IgG and IgM, and 1% for IgA); interestingly, no SARS-CoV-2-infected patients with IBD developed symptomatic COVID-19 (Łodyga et al., 2021). Further studies reported similar results, confirming that SARS-CoV-2 seroprevalence among individuals with IBD is closely comparable to that in subjects without IBD (Norsa et al., 2020; Bertè et al., 2021).

## Gut Microbiota in IBD

Gut microbiota plays a key role in health and disease; it actively impacts multiple host systems and organs. A balanced gut microbial ecosystem with high biodiversity is associated with the beneficial effects of a myriad of symbiotic interactions between intra- and inter-microbial species, genera, families, phyla, and between microbes and host systems and organs, such as the immune system (Zheng et al., 2020), the brain, and the lung (Morais et al., 2021; Sencio et al., 2021). Conversely, perturbations in gut microbial communities, namely, dysbiosis, induce detrimental effects on these networks and are associated with diseases (Durack and Lynch, 2019). Gut dysbiosis can be defined as the loss of the overall microbial biodiversity with the imbalance between beneficial commensal and opportunistic pathogens, resulting in excessive production of pro-inflammatory mediators (Wei et al., 2021). A large body of literature investigated and evaluated extensively gut dysbiosis in individuals with IBD; the most frequently observed alterations are the overgrowth of pro-inflammatory bacterial species (e.g., *Escherichia coli*) associated with the shortfall of anti-inflammatory species (e.g., *Faecalibacterium prausnitzii*). The latter are involved in the generation of short-chain fatty acids (SCFAs), namely, butyrate, propionate, and acetate (Zuo and Ng, 2018; Khan et al., 2019; Aldars-García et al., 2021; Alshehri et al., 2021). Regrettably, data on gut microbiota composition are partially heterogeneous between studies, and results could be categorized as (a) fully concordant between studies; (b) roughly concordant with some exceptions; (c) discordant between studies. Table 1 recapitulates the most relevant data on gut dysbiosis in IBD, obtained from a great proportion of available studies from the literature (Favier et al., 1997; Seksik et al., 2003; Macfarlane et al., 2004; Martin et al., 2004; Ott et al., 2004; Gophna et al., 2006; Manichanh et al., 2006; Scanlan et al., 2006; Frank et al., 2007; Andoh et al., 2009; Kang et al., 2010; Rehman et al., 2010; Schwiertz et al., 2010; Willing et al., 2010; Joossens et al., 2011; Mondot et al., 2011; Rausch et al., 2011; Walker et al., 2011; Michail et al., 2012; Morgan et al., 2012; Nemoto et al., 2012; Vigsnæs et al., 2012; Fujimoto et al., 2013; Kabeerdoss et al., 2013, 2015; Kumari et al., 2013; Prideaux et al., 2013; Sha et al., 2013; Tong et al., 2013; Varela et al., 2013; Gevers et al., 2014; Hedin et al., 2014; Machiels et al., 2014; Walters et al., 2014; Wang et al., 2014; Hoarau et al., 2016; Jacobs et al., 2016; Mar et al., 2016; Takahashi et al., 2016; Chen et al., 2017; Halfvarson et al., 2017; Pascal et al., 2017; Santoru et al., 2017; Sokol et al., 2017; Vrakas et al., 2017; Zhang et al., 2017, 2021; de Meij et al., 2018; Laserna-Mendieta et al., 2018; Nishino et al., 2018; Franzosa et al., 2019; Heidarian et al., 2019; Lloyd-Price et al., 2019; Yilmaz et al., 2019; Alam et al., 2020; Ryan et al., 2020; Shahir et al., 2020; Clooney et al., 2021). No specific pattern of dysbiosis in patients with IBD has been definitively established; nevertheless, there is a broad agreement between studies on the imbalance of gut bacterial abundance in IBD. In particular, most studies report the depletion of *Clostridium* genus, *C. leptum* (cluster IV), *C. coccoides* (cluster XIVa) groups, *F. prausnitzii*, *E. rectale*, *R. bromii* species, *Ruminococcaceae*, *Lachnospiraceae* families, and the overgrowth of *Enterococcus* and *Fusobacterium* genera, *E. coli* and *F. nucleatum* species, *Enterobacteriaceae*, *Veillonellaceae* families. Controversial results may derive from many variables affecting gut microbiota composition, including the pre-existence of chronic diseases, the intensive therapeutic treatment in critically ill patients, especially with antibiotics to prevent secondary bacterial infections, and sudden and radical changes to eating habits (Table 2). Gut microbiome composition in IBD is strongly influenced by complex interactions between microbial communities and genetically altered host functional pathways (Huang et al., 2014; Knights et al., 2014). In CD, gut dysbiosis is more pronounced than in UC and is marked by a lower microbial diversity, a more altered microbiome composition, and a more unstable microbial community (Pascal et al., 2017). Microbial diversity and abundance significantly differ between feces and gut mucosa, as reported in early studies (Lepage et al., 2005; Gillevet et al., 2010; Morgan et al., 2012) and confirmed in more recently published papers (Lo Presti et al., 2019; Ryan et al., 2020). In IBD, inflammation alters the mucosal barrier inducing bacterial translocation; in patients with CD, bacterial translocation is revealed by the increase in several bacterial families within the submucosa compared to the corresponding superjacent mucosa at the advancing disease margin (Chiodini et al., 2016). Mucosal and fecal microbiome differences may explain, at least in part, some discrepancies between studies; for example, the increase in fecal *F. prausnitzii* (Table 1) corresponds to the decreased proportion of this bacterium at the mucosal surface (Walters et al., 2014). Differences in the abundance of various bacterial species and families (e.g., *Lactobacilli*, *C. leptum group*, *E. coli*, and *F. prausnitzii*) were observed between ulcerated (inflamed) and non-ulcerated (non-inflamed) mucosa (Zhang et al., 2007; Li et al., 2012) as well as between patients with high clinical activity indexes and/or sigmoidoscopy scores and patients with low clinical activity indexes and/or sigmoidoscopy scores (Fite et al., 2013). Other studies reported no difference in microbiota composition and enrichment between inflamed and non-inflamed mucosa (Kabeerdoss et al., 2015; Nishino et al., 2018). The abundance of some bacteria, such as the genus of *Faecalibacterium* and the family of *Enterobacteriaceae*, significantly differs between ileal CD and colonic CD (Dicksved et al., 2008; Naftali et al., 2016); for example, *F. prausnitzii* is markedly reduced in CD localized in the ileum compared with colonic localization (Willing et al., 2010). Interestingly, in current smokers with CD, the abundance of *Bacteroides–Prevotella* genera is higher than in non-smokers with CD (Benjamin et al., 2012a). Significant differences in several microbial taxa can be observed between young adults with IBD and IBD adults aged 60 years or older; in particular, *Bifidobacterium* genus decrease with age, and *Bacteroides* genus increase with age, probably reflecting body mass index and diet changes over time (Morgan et al., 2012). Researchers have a unanimous consensus on the *E. coli* overgrowth in IBD. *E. coli* overgrowth has been found in children with severe IBD (Schwiertz et al., 2010; Michail et al., 2012; Gevers et al., 2014; de Meij et al., 2018) and in adults with CD (Mondot et al., 2011). In patients with CD, the high prevalence of *E. coli* strictly adhering to the ileal mucosa has led to the identification of a new group of *E. coli* strains (Nadalian et al., 2021). This pathogenic group, called adherent-invasive *E. coli* (AIEC), has the ability to adhere and colonize enterocytes as well as to internalize into macrophages and replicate within their cytoplasm, inducing the release of tumor necrosis factor-α (TNF-α) and the cytotoxic response of Th17 and CD8^+^ (Lee et al., 2019). Thus, AIEC is involved in the pathogenesis of IBD, specifically CD (Palmela et al., 2018; Chervy et al., 2020), by promoting inflammatory diseases that originated from the adaptative evolution of the genome (Nash et al., 2010; Ellermann et al., 2015). The recognition of AIEC is unusual in patients with UC; rather, UC is associated with the intestinal enrichment of a heterogeneous, diarrheagenic group of *E. coli* strains, termed diffusely adherent *E. coli* (DAEC); this group was found expressed not only in children and young adults with UC but even in those with CD (Walczuk et al., 2019).

**Table 1:** Gut dysbiosis in patients with IBD and COVID-19 compared with healthy subjects (s, stool sample; m, mucosal biopsy; e, endoscopic lavage).

Bacterial taxa	Inflammatory bowel disease (IBD)		Coronavirus Disease 2019 (COVID-19)	
	Enriched	Underrepresented	Enriched	Underrepresented
**Firmicutes**				
*Clostridium*	Morgan et al., 2012 (s,m)	Gophna et al., 2006 (m), Michail et al., 2012 (s), Tong et al., 2013 (e), Walters et al., 2014 (s), Gevers et al., 2014 (m), Chen et al., 2017 (s), Nishino et al., 2018 (m)	Zuo et al., 2020 (s), Tao et al., 2020 (s) [*C. hathewayi*]	
*Clostridium cluster IV (C. leptum)*		Seksik et al., 2003 (s), Scanlan et al., 2006 (s), Manichanh et al., 2006 (s), Andoh et al., 2009 (s), Schwiertz et al., 2010 (s), Mondot et al., 2011 (s), Morgan et al., 2012 (s,m), Kabeerdoss et al., 2013 (s), Sha et al., 2013 (s), Kumari et al., 2013 (s), Fujimoto et al., 2013 (s), Wang et al., 2014 (s,m), Hedin et al., 2014 (s), Kabeerdoss et al., 2015 (m), Vrakas et al., 2017 (m), Laserna-Mendieta et al., 2018 (s), Shahir et al., 2020 (m)		Tang et al., 2020 (s), Yeoh et al., 2021 (s) [during antibiotic therapy]
*Clostridium cluster XIVa (C. coccoides)*		Seksik et al., 2003 (s), Scanlan et al., 2006 (s), Andoh et al., 2009 (s), Schwiertz et al., 2010 (s), Joossens et al., 2011 (s), Morgan et al., 2012 (s,m), Nemoto et al., 2012 (s), Sha et al., 2013 (s), Kumari et al., 2013 (s), Prideaux et al., 2013 (m), Hedin et al., 2014 (s), Machiels et al., 2014 (s), Kabeerdoss et al., 2015 (m), Vrakas et al., 2017 (m), Clooney et al., 2021 (s)		
*F. prausnitzii*	Walters et al., 2014 (s)	Frank et al., 2007 (m), Schwiertz et al., 2010 (s), Willing et al., 2010 (s), Rehman et al., 2010 (m), Mondot et al., 2011 (s), Joossens et al., 2011 (s), Rausch et al., 2011 (m), Morgan et al., 2012 (s,m), Fujimoto et al., 2013 (s), Varela et al., 2013 (s), Tong et al., 2013 (e), Kabeerdoss et al., 2013 (s), Kumari et al., 2013 (s), Prideaux et al., 2013 (m), Machiels et al., 2014 (s), Wang et al., 2014 (s,m), Hedin et al., 2014 (s), Gevers et al., 2014 (m), Takahashi et al., 2016 (s), Mar et al., 2016 (s), Hoarau et al., 2016 (s), Jacobs et al., 2016 (s), Sokol et al., 2017 (s), Vrakas et al., 2017 (m), Pascal et al., 2017 (s), Halfvarson et al., 2017 (s), Santoru et al., 2017 (s), Laserna-Mendieta et al., 2018 (s), Franzosa et al., 2019 (s), Lloyd-Price et al., 2019 (s,m), Yilmaz et al., 2019 (m), Heidarian et al., 2019 (s), Ryan et al., 2020 (m), Clooney et al., 2021 (s), Zhang et al., 2021 (s,m)		Tang et al., 2020 (s), Zuo et al., 2020 (s) [during antibiotic therapy], Tao et al., 2020 (s), Yeoh et al., 2021 (s), Gaibani et al., 2021 (s)
*E. rectale*		Macfarlane et al., 2004 (m), Mondot et al., 2011 (s), Kumari et al., 2013 (s), Gevers et al., 2014 (m), Kabeerdoss et al., 2015 (m), Franzosa et al., 2019 (s), Clooney et al., 2021 (s)		Tang et al., 2020 (s), Zuo et al., 2020 (s) [during antibiotic therapy], Yeoh et al., 2021 (s)
*Enterococcus*	Macfarlane et al., 2004 (m), Kang et al., 2010 (s), Mondot et al., 2011 (s), Nemoto et al., 2012 (s), Tong et al., 2013 (e), Gevers et al., 2014 (m), Takahashi et al., 2016 (s), Mar et al., 2016 (s), Zhang et al., 2017 (s), Pascal et al., 2017 (s), Franzosa et al., 2019 (s)		Tang et al., 2020 (s), Wu et al., 2021 (s), Gaibani et al., 2021 (s)	
*Ruminococcaceae*		Morgan et al., 2012 (s,m), Hedin et al., 2014 (s), Mar et al., 2016 (s), Halfvarson et al., 2017 (s), Sokol et al., 2017 (s), Zhang et al., 2021 (s,m), Nishino et al., 2018 (m),		Gu et al., 2020 (s), He et al., 2021 (s), Gaibani et al., 2021 (s)
*R. bromii*		Frank et al., 2007 (m), Mondot et al., 2011 (s), Prideaux et al., 2013 (m), Hoarau et al., 2016 (s), Sokol et al., 2017 (s), Nishino et al., 2018 (m), Ryan et al., 2020 (m),		Yeoh et al., 2021 (s)
*R. gnavus*	Willing et al., 2010 (s), Joossens et al., 2011 (s), Machiels et al., 2014 (s), Hoarau et al., 2016 (s), Sokol et al., 2017 (s), Nishino et al., 2018 (m), Franzosa et al., 2019 (s), Lloyd-Price et al., 2019 (s,m), Yilmaz et al., 2019 (m), Ryan et al., 2020 (m), Clooney et al., 2021 (s),	Frank et al., 2007 (m), Gevers et al., 2014 (m)	Yeoh et al., 2021 (s)	
*Lachnospiraceae*	Alam et al., 2020 (s)	Frank et al., 2007 (m), Rausch et al., 2011 (m), Kumari et al., 2013 (s), Prideaux et al., 2013 (m), Mar et al., 2016 (s), Chen et al., 2017 (s), Sokol et al., 2017 (s), Nishino et al., 2018 (m), Yilmaz et al., 2019 (m), Ryan et al., 2020 (m),		Zuo et al., 2020 (s) [during antibiotic therapy], Gu et al., 2020 (s), Zuo et al., 2021 (s), Gaibani et al., 2021 (s), He et al., 2021 (s), Wu et al., 2021 (s)
*R. hominis*		Tong et al., 2013 (e), Machiels et al., 2014 (s), Franzosa et al., 2019 (s), Lloyd-Price et al., 2019 (s,m)		
*D. forminigenerans*		Franzosa et al., 2019 (s), Zhang et al., 2021 (s,m)		Yeoh et al., 2021 (s) [during antibiotic therapy]
*Lactobacillus*	Willing et al., 2010 (s), Kang et al., 2010 (s), Fujimoto et al., 2013 (s), Wang et al., 2014 (s,m), Kabeerdoss et al., 2015 (m), Zhang et al., 2021 (s,m)	Ott et al., 2004 (m), Frank et al., 2007 (m), Rausch et al., 2011 (m), Vigsnaes et al., 2012 (s), Sha et al., 2013 (s), Vrakas et al., 2017 (m), Zhang et al., 2017 (s)	Gu et al., 2020 (s), Tao et al., 2020 (s), Yeoh et al., 2021 (s), Wu et al., 2021 (s) Gaibani et al., 2021 (s)	Tang et al., 2020 (s)
*Veillonellaceae*	Macfarlane et al., 2004 (m), Michail et al., 2012 (s), Gevers et al., 2014 (m), Santoru et al., 2017 (s), Lloyd-Price et al., 2019 (s,m), Alam et al., 2020 (s), Ryan et al., 2020 (m)		Gu et al., 2020 (s), Gaibani et al., 2021 (s)	
**Proteobacteria**				
*Enterobacteriaceae*	Seksik et al., 2003 (s), Frank et al., 2007 (m), Andoh et al., 2009 (s), Michail et al., 2012 (s), Nishino et al., 2018 (m), Alam et al., 2020 (s), Ryan et al., 2020 (m), Shahir et al., 2020 (m)		Tang et al., 2020 (s)	
*E. coli*	Martin et al., 2004 (m), Gophna et al., 2006 (m), Schwiertz et al., 2010 (s), Willing et al., 2010 (s), Rehman et al., 2010 (m), Mondot et al., 2011 (s), Michail et al., 2012 (s), Morgan et al., 2012 (s,m), Tong et al., 2013 (e), Sha et al., 2013 (s), Gevers et al., 2014 (m), Wang et al., 2014 (s,m), Kabeerdoss et al., 2015 (m), Hoarau et al., 2016 (s), Takahashi et al., 2016 (s), Zhang et al., 2017 (s), Pascal et al., 2017 (s), Santoru et al., 2017 (s), Vrakas et al., 2017 (m), Chen et al., 2017 (s), de Meij et al., 2018 (s), Franzosa et al., 2019 (s), Lloyd-Price et al., 2019 (s,m), Zhang et al., 2021 (s,m)			
*Shigella*	Willing et al., 2010 (s), Kang et al., 2010 (s), Morgan et al., 2012 (s,m)			
*P. mirabilis*	Zhang et al., 2021 (s,m)			
*Sutterella*	Frank et al., 2007 (m), Michail et al., 2012 (s), Pascal et al., 2017 (s)	Rausch et al., 2011 (m)	Yeoh et al., 2021 (s) [during antibiotic therapy]	
**Fusobacteria**				
*Fusobacterium*	Michail et al., 2012 (s), Alam et al., 2020 (s), Zhang et al., 2021 (s,m)			
*F. nucleatum*	Gevers et al., 2014 (m), Pascal et al., 2017 (s), Santoru et al., 2017 (s), Clooney et al., 2021 (s)			
**Bacteroidetes**				
*Bacteroides*	Andoh et al., 2009 (s), Walker et al., 2011 (m), Wang et al., 2014 (s,m), Kabeerdoss et al., 2015 (m), Hoarau et al., 2016 (s), Vrakas et al., 2017 (m)	Seksik et al., 2003 (s), Ott et al., 2004 (m), Rehman et al., 2010 (m), Nemoto et al., 2012 (s), Sha et al., 2013 (s), Fujimoto et al., 2013 (s), Gevers et al., 2014 (m), Takahashi et al., 2016 (s), Mar et al., 2016 (s), Sokol et al., 2017 (s), Heidarian et al., 2019 (s)	Zuo et al., 2020 (s), Yeoh et al., 2021 (s) [*B. dorei* during antibiotic therapy], He et al., 2021 (s)	Tao et al., 2020 (s), Zuo et al., 2021 (s), Wu et al., 2020 (s) [during patient’s admission in ICU]
*B. fragilis*	Gophna et al., 2006 (m), Walters et al., 2014 (s), Ryan et al., 2020 (m), Shahir et al., 2020 (m)	Manichanh et al., 2006 (s), Scanlan et al., 2006 (s), Macfarlane et al., 2004 (m), Kang et al., 2010 (s), Sha et al., 2013 (s), Jacobs et al., 2016 (s), de Meij et al., 2018 (s)		
*B. vulgatus*		Macfarlane et al., 2004 (m), Gevers et al., 2014 (m), Ryan et al., 2020 (m)	Yeoh et al., 2021 (s)	
*B. ovatus*		Frank et al., 2007 (m), Macfarlane et al., 2004 (m), Santoru et al., 2017 (s), Shahir et al., 2020 (m)	Yeoh et al., 2021 (s)	
*Prevotellaceae*	Manichanh et al., 2006 (s), Andoh et al., 2009 (s), Walker et al., 2011 (m), Kabeerdoss et al., 2015 (m), Alam et al., 2020 (s)	Seksik et al., 2003 (s), Ott et al., 2004 (m), Kang et al., 2010 (s), Rausch et al., 2011 (m), Sha et al., 2013 (s), Fujimoto et al., 2013 (s), Prideaux et al., 2013 (m), Hedin et al., 2014 (s), Takahashi et al., 2016 (s), Mar et al., 2016 (s), Hoarau et al., 2016 (s), Santoru et al., 2017 (s), Sokol et al., 2017 (s), Nishino et al., 2018 (m), Heidarian et al., 2019 (s)		Gaibani et al., 2021 (s)
*Alistipes*	Walker et al., 2011 (m), Rausch et al., 2011 (m), Shahir et al., 2020 (m)	Frank et al., 2007 (m), Willing et al., 2010 (s), Mondot et al., 2011 (s), Gevers et al., 2014, Ryan et al., 2020 (m), (m), Chen et al., 2017 (s), Sokol et al., 2017 (s), Halfvarson et al., 2017 (s), Nishino et al., 2018 (m), de Meij et al., 2018 (s), Franzosa et al., 2019 (s), Lloyd-Price et al., 2019 (s,m), Shahir et al., 2020 (m)		Zuo et al., 2021 (s), Yeoh et al., 2021 (s) [*A. putredinis* during antibiotic therapy]
**Verrucomicrobia**				
*A. muciniphila*		Vigsnaes et al., 2012 (s), Jacobs et al., 2016 (s), Santoru et al., 2017 (s), de Meij et al., 2018 (s)	Gaibani et al., 2021 (s)	
*Actinobacteria*				
*Bifidobacterium*	Willing et al., 2010 (s), Wang et al., 2014 (s,m), Takahashi et al., 2016 (s),	Favier et al., 1997 (s), Seksik et al., 2003 (s), Schwiertz et al., 2010 (s), Kang et al., 2010 (s), Sha et al., 2013 (s), Sokol et al., 2017 (s), Zhang et al., 2017 (s), Vrakas et al., 2017 (m), Yilmaz et al., 2019 (m), Alam et al., 2020 (s)	Tao et al., 2020 (s), Wu et al., 2021 (s)	Tang et al., 2020 (s)
*B. bifidum*		Macfarlane et al., 2004 (m), Mondot et al., 2011 (s), Gevers et al., 2014 (m)		
*B. adolescentis*	Jacobs et al., 2016 (s), Mar et al., 2016 (s)	Macfarlane et al., 2004 (m), Joossens et al., 2011 (s), Hedin et al., 2014 (s), Gevers et al., 2014 (m), Machiels et al., 2014 (s)		Yeoh et al., 2021 (s)
*Collinsella*	Willing et al., 2010 (s)	Joossens et al., 2011 (s), Santoru et al., 2017 (s), Pascal et al., 2017 (s), Nishino et al., 2018 (m),	Zuo et al., 2021 (s), Gaibani et al., 2021 (s)	Yeoh et al., 2021 (s), Wu et al., 2021 (s)

**Table 2:** Main factors affecting the variability of the results between published studies on gut microbiota composition in IBD.

1. Host genetic polymorphisms and gene expression
2. Mucosal immune system interactions (e.g., with Treg/Th17, PRRs, TLRs, and NLRs)
3. Disease phenotype based on clinical activity indices and sigmoidoscopy scores
4. Type of disease (e.g., Crohn’s Disease and Ulcerative Colitis)
5. Type of biological sample (e.g., stool, endoscopic biopsies, or resection specimens)
6. Site of the biopsy sampling (e.g., terminal ileum and large bowel)
7. Host demographics (e.g., gender and aging)
8. Environmental stimuli and patient’s life style (e.g., smoking)
9. Diet and medications (e.g., antibiotics)
10. Inter-individual variability between patients
11. Methods for the microbiome analysis (e.g., fluorescence *in situ*, terminal restriction fragment length polymorphism, 16S rDNA sequencing, and whole-genome sequence)

## Gut Microbiota in SARS-CoV-2 Infection

Although the lung is considered the main entry route for SARS-CoV-2, the gastrointestinal tract is equally a key target of the virus (Xiao et al., 2020; Qian et al., 2021). Indeed, the brush border of human enterocytes exhibits the highest expression of the SARS-CoV-2 receptor angiotensin-converting enzyme 2 (ACE2; Qi et al., 2020); even the transmembrane serine protease 2 (TMPRSS2), mediating the entry of SARS-CoV-2, is expressed on the luminal surface of enterocytes from ileum and colon as well as on the epithelial and gland cells of the esophagus (Knyazev et al., 2021). Thus, it is not surprising that SARS-CoV-2 infects human gut enterocytes (Lamers et al., 2020), promoting gut mucosal inflammatory infiltration with activated immune cells and cytokines (Lehmann et al., 2021). These findings support (a) the frequently observed gastrointestinal symptoms in patients with COVID-19, including severe abdominal pain, diarrhea, nausea, and vomiting (Devaux et al., 2021); (b) the persistence of viral RNA in patient’s stool even after the virus clearance from oropharyngeal swab (Morone et al., 2020); (c) the likelihood of SARS-CoV-2 transmission by the fecal–oral route (Cheung et al., 2020; Guo et al., 2021); (d) gut dysbiosis in asymptomatic infected individuals and patients with COVID-19 (Yamamoto et al., 2021). SARS-CoV-2 gut colonization significantly alters the gut microbial ecosystem, leading to dysbiosis; on the other hand, gut dysbiosis, due to aging, unhealthy lifestyle habits, and pre-existing chronic diseases (e.g., hypertension, type-2 diabetes, autoimmune diseases, and metabolic syndrome), is a key risk factor for developing COVID-19 (Magalhães et al., 2021). In hospitalized patients with COVID-19, the reduction in gut microbiota diversity, the depletion of beneficial bacterial symbionts, and the enrichment of opportunistic pathogens closely correlate with the host immune response and, in turn, with the disease severity and the clinical outcome (Villapol, 2020). The variety of the therapeutic treatment for COVID-19 may impact changes in gut microbiome composition, as highlighted in a recent review (Aktas and Aslim, 2021). Gut dysbiosis continues after the clearance of the viral RNA from the upper respiratory tract and the resolution of clinical symptoms. These associations result from complex interactions between the gut and the lung microbiota, the so-called gut–lung axis (de Oliveira et al., 2021). In particular, a balanced gut microbial ecosystem enhances the pulmonary defense against viral infections, for example, by stimulating the lung’s synthesis of type I interferons (Cyprian et al., 2021). Conversely, gut dysbiosis negatively influences the progression of the viral infection, COVID-19 development, and patient outcome (Hussain et al., 2021).

Since SCFAs inhibit the overgrowth of opportunistic pathogens, activate the adaptive immune response by enhancing antiviral immunity, and contribute to maintaining the integrity of the intestinal mucosal barrier, the depletion of SCFAs producer bacteria is closely associated with COVID-19 severity and adverse outcome; therefore, the number of SCFAs producer bacteria could predict the severity of the disease (Tang et al., 2020). Based on the observation that the *Enterococcus*/*Enterobacteriaceae* ratio is altered in approximately 74% of patients with severe/critical COVID-19, being significantly increased in non-survivors compared with survivors, it was proposed that this index may be useful to predict death in critically ill patients (Tang et al., 2020). However, a strong limitation may be the heterogeneity of this ratio. *Enterococcus* is a genus belonging to the *Enterococcaceae* family (*Firmicutes* phylum), while *Enterobacteriaceae* family belongs to *Proteobacteria* phylum. Despite the limited number of studies on the gut microbiome in patients with COVID-19, the pattern of gut dysbiosis associated with the disease has been partially defined (Table 1). When compared with non-infected individuals, gut dysbiosis in patients with COVID-19 is marked by the depletion of *C. leptum* (cluster IV) group, *F. prausnitzii*, and *E. rectale* species, *Ruminococcaceae*, and *Lachnospiraceae* families, in conjunction with the overgrowth of *Enterococcus* genus, *Veillonellaceae*, and *Enterobacteriaceae* families (Tang et al., 2020; Tao et al., 2020; Zuo et al., 2020, 2021; Gaibani et al., 2021; He et al., 2021; Wu et al., 2021; Yeoh et al., 2021). The abundance of *Coprobacillus*, *C. ramosum*, *C. hathewayi* (Zuo et al., 2020), and *Enterococcus* (Gaibani et al., 2021) was found positively correlated with COVID-19 severity; conversely, an inverse correlation was observed between the disease severity and the abundance of *C. leptum* (cluster IV) group, *Lactobacillus*, *Bifidobacterium*, *C. butyricum* (Tang et al., 2020), *Bilophila*, *Citrobacter* (Tao et al., 2020), *Bacteroides* (Gaibani et al., 2021), *F. prausnitzii*, *E. rectale* (Tang et al., 2020; Yeoh et al., 2021), *B. bifidum*, and *B. adolescentis* (Yeoh et al., 2021). In addition, the abundance of some microbial taxa, including *Erysipelotrichaceae bacterium 2_2_44A* (Zuo et al., 2020), *P. copri*, *E. dolichum* (Wu et al., 2021), *C. aerofaciens*, *C. tanakaei*, *S. infantis* (Zuo et al., 2021) positively correlates with the fecal SARS-CoV-2 load. Conversely, the abundance of *B. dorei*, *B. thetaiotaomicron*, *B. massiliens*, *B. ovatus* (Zuo et al., 2020), *S. anginosus*, *Dialister*, *Alistipes*, *Ruminococcus*, *C. citroniae*, *Bifidobacterium*, *Haemophilus*, *H. parainfluenzae* (Wu et al., 2021), *P. merdae*, *B. stercoris*, *A. onderdonkii*, and *Lachnospiraceae bacterium 1_1_57FAA* (Zuo et al., 2021) was inversely correlated with the fecal viral load. The increased gut colonization of bacteria usually resident in the oral and respiratory tracts, such as *Actinomyces* (Gu et al., 2020; Zuo et al., 2020; Gaibani et al., 2021; Wu et al., 2021) and *Granulicatella* (Wu et al., 2021), is associated with COVID-19 and its severity, confirming the active interchange between the gut, oral, and respiratory tract microbiota. The close relationship between gut microbiota and the immune-mediate response to SARS-CoV-2 infection and COVID-19 progression and outcome is supported by the correlation between the abundance of some gut microbial taxa and biomarkers of inflammation. For example, in critical patients with COVID-19, a negative correlation was observed between serum C-reactive protein (CRP) levels and the gut abundance of *C. butyricum* and *F. prausnitzii* (Tang et al., 2020). Fecal inflammatory cytokine IL-18 concentration was positively correlated with the abundance of *Peptostreptococcus*, *Fusobacterium*, and *Citrobacter* in patients with COVID-19 (Tao et al., 2020). Similarly, *B. dorei* and *A. muciniphila* abundance was found positively correlated with the serum level of IL-1β, IL-6, and C-X-C motif ligand 8 (CXCL8), whereas *F. prausnitzii*, *E. rectale*, and *B. adolescentis* were negatively correlated with serum level of TNF-α, IL-10, C-C motif ligand 2 (CCL2), and CXCL10 (Yeoh et al., 2021).

## Metabolomics in IBD and COVID-19

Metabolomics is an evolving “omic” discipline allowing the identification and the quantification of endogenous and exogenous products of the cellular metabolism, namely, metabolites, within a biological system in a high-throughput manner (Liu and Locasale, 2017). The set of metabolites recognizable in a biological matrix is called metabolome or metabolic profile; it is a highly personalized readout of the current metabolism and metabolic activity that occurred in the past (Zamboni et al., 2015). Qualitative and quantitative data on metabolites reveal basic information on changes and perturbations of metabolic pathways deriving from interactions between genome, environment, microbiome, nutrients, and the intake of drugs and toxicants in health and disease states (Ashrafian et al., 2020). An updated PubMed literature search, querying the keyword metabolomics, results in approximately 26,200 studies, including *in vitro* experimental studies, studies on animal models, and clinical studies on patients with various diseases (Kang et al., 2021). Approximately 200 studies used the metabolomic approach in patients with IBD (Gallagher et al., 2021) and 25 in patients with COVID-19 (Mussap and Fanos, 2021). The most relevant findings are reported below.

### Tryptophan Metabolism

**Figure 1:**
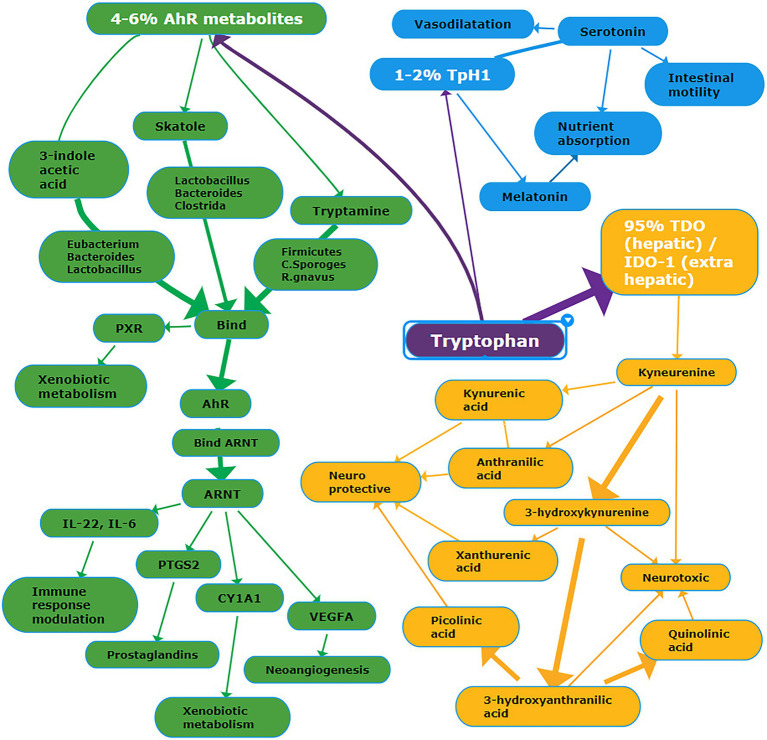
Schema summarizing the main metabolic pathways deriving from tryptophan. Details are reported in the text. Abbreviations: PXR, Pregnane X Receptor; AhR, Aryl Hydrocarbon Receptor; ARNT, AhR Nuclear Translocator protein; PTGS2, Prostaglandin G/H Synthase 2; CYP1A1, Cytochrome P450 1A1; VEGFA, vascular endothelial growth factor A; TpH1, tryptophan hydroxylase-1; TDO, tryptophan dioxygenase; IDO-1, indoleamine 2,3-dioxygenase-1. White font = name of the metabolic pathway; purple background = tryptophan pathway; yellow background = TDO/IDO-1 pathway; green background = AhR metabolic pathway; blue background = TpH1 pathway.

Tryptophan (TRP) is an essential amino acid mainly derived from the diet and involved in serotonin, melatonin, and niacin biosynthesis, as detailed in Figure 1. More than 95% of TRP is metabolized along the kynurenine pathway; in the liver, the enzyme tryptophan dioxygenase (TDO) converts tryptophan into kynurenine. In the brain and the immunocompetent cells, the conversion is catalyzed by indoleamine 2,3-dioxygenase (IDO-1 and 2; Gao et al., 2018; Agus et al., 2018). Kynurenine can be converted either into neurotoxic metabolites, namely, 3-hydroxykynurenine, 3-hydroxyanthranilic acid, quinolinic acid, or neuroprotective metabolites, such as kynurenic acid, anthranilic acid, xanthurenic acid, and picolinic acid (Savitz, 2020). Depending on gut microbiota composition, approximately 4–6% of tryptophan is converted into various intermediates. For example, the prevalence of *Clostridium sporogenes* and *Ruminococcus gnavus* spp. originates tryptamine (Williams et al., 2014), whereas the prevalence of *Lactobacillus*, *Bacteroides,* and *Clostridium* genera originates indole derivatives (Roager and Licht, 2018). Among bacterial metabolites, indoles play a crucial role in the regulation of gastrointestinal barrier function and integrity by their binding with the pregnane X receptor (PXR), also known as steroid and xenobiotic receptor (SXR; Venkatesh et al., 2014; Oladimeji and Chen, 2018). Finally, 1–2% tryptophan is converted into serotonin (5-hydroxytryptamine), a neurotransmitter and key regulator of intestinal secretion and motility. More than 90% of serotonin is synthesized by the rate-limiting enzyme tryptophan hydroxylase (Tph/TPH) 1 within enterochromaffin cells of the gut (Stasi et al., 2019); notably, gut microbiota may considerably affect serotonergic regulation *via* microbiota-derived SCFA (Reigstad et al., 2015).

Tryptophan and indole-3-acetic are decreased in the blood of patients with IBD (Table 3; Ooi et al., 2011; Kohashi et al., 2014; Nikolaus et al., 2017; Bosch et al., 2018; Lai et al., 2019). Conversely, kynurenine and quinolinic acid are increased (Forrest et al., 2002; Yau et al., 2014; Nikolaus et al., 2017; Whiley et al., 2019). In the urine and stool of patients with IBD, tryptophan is increased (Schicho et al., 2012; Bosch et al., 2018). Kynurenic acid blood levels were increased in IBD (Forrest et al., 2002); similarly, picolinic acid was increased (Yau et al., 2014). More recently, kynurenic acid and picolinic acid were found decreased in patients with IBD (Nikolaus et al., 2017; Whiley et al., 2019). These alterations seem to be closely related to gut dysbiosis (de Meij et al., 2018), promoting the massive activation of pro-inflammatory cytokines (e.g., INF-γ and TNF-α), and the upregulation of the IDO expression (Wu et al., 2018). Gut dysbiosis significantly affects the conversion of tryptophan into indole derivatives, such as indole-3 acetic acid and indole-3-acetaldehyde. Low blood levels of indole-3-acetic acid have been associated with the overgrowth of *Clostridium* and *Lactobacillus* genera; both bacterial genera decarboxylate indole-3-acetic acid in 3-methylindole, also known as skatole. As a result, indole-3-acetic acid is metabolized with the concomitant accumulation of skatole. In patients with IBD, skatole blood levels were significantly increased (Lai et al., 2019). Most indole derivatives are ligands of the Aryl Hydrocarbon Receptor (AHR), a cytosolic ligand-dependent transcription factor widely expressed by cells of the immune system and involved in antimicrobial activity and gut immune homeostasis (Stockinger et al., 2014; Lamas et al., 2018). The indole derivatives-induced AHR activation promotes the local synthesis of the anti-inflammatory cytokine IL-22 by the innate lymphoid cells (Monteleone et al., 2011; Qiu et al., 2012; Yang et al., 2020); IL-22 protects the mucosa integrity against fungal infection by Candida albicans, commonly observed in patients with IBD (Sokol et al., 2017; Gronke et al., 2019). In healthy subjects with gut eubiosis, the activation of AHR modulates local IL-22 production; conversely, in IBD patients, the decrease of indole-3 acetic acid due to the imbalance of gut flora reduces the AHR activity and hence IL-22 synthesis, as observed in an animal model (Lamas et al., 2016). In patients with COVID-19, most metabolomics-based studies reported a significant decrease in blood tryptophan level in conjunction with the significant increase of kynurenine, kynurenic acid, and downstream metabolites of the kynurenine pathway (Barberis et al., 2020; Fraser et al., 2020; Thomas et al., 2020; Lawler et al., 2021). The decrease of tryptophan was inversely correlated with biomarkers of inflammation, such as IL-6 and CRP (Thomas et al., 2020). The increase of kynurenine is significantly associated with the increase of several cytokines, including interferon γ-induced protein 10 (IP-10), the mitogen-inducible cytokine macrophage inflammatory protein-1 β (MIP-1-β), also known as CCL4, TNF-α, interleukin-1 receptor antagonist (IL-1RA), IL-7, IL-18, and IL-8 (Lawler et al., 2021). Controversial results on indole-3-acetic acid in patients with COVID-19 have been published. On the one hand, it was found decreased (Lawler et al., 2021), and this finding remains unclear. A possible explanation may be the decrease of indole-3 acetic acid produced by the host because of the upregulation of the kynurenine pathway. This assumption is plausible, taking into account that a fraction of indole-3 acetic acid is produced by mammalian cells (Zhang et al., 2020). On the other hand, indole-3 acetic acid has been found increased (Blasco et al., 2020); this finding is coherent with gut dysbiosis marked by the prevalence of bacteria converting tryptophan into indoles.

**Table 3:** Changes in the tryptophan metabolism in IBD and in COVID-19.

Metabolite	Inflammatory bowel disease (IBD)		Coronavirus disease 2019 (COVID-19)	
	Trend	Ref.	Trend	Ref.
Tryptophan (blood)	Decreased	Ooi et al., 2011; Kohashi et al., 2014; Nikolaus et al., 2017; Bosch et al., 2018	Decreased	Barberis et al., 2020; Thomas et al., 2020; Lawler et al., 2021
Tryptophan (urine)	Increased	Schicho et al., 2012		
Tryptophan (stool)	Increased	Bosch et al., 2018		
Kynurenine (blood)	Increased	Forrest et al., 2002; Nikolaus et al., 2017; Whiley et al., 2019	Increased	Fraser et al., 2020; Thomas et al., 2020; Lawler et al., 2021
Quinolinic acid (blood)	Increased	Yau et al., 2014; Nikolaus et al., 2017;	Increased	Lawler et al., 2021
Kynurenic acid (blood)	Increased	Forrest et al., 2002	Increased	Thomas et al., 2020 (only in patients with high levels of interleukine-6)
	Decreased	Nikolaus et al., 2017; Whiley et al., 2019 (statistically not significant)		
Indole-3-acetic acid (blood)	Decreased	Lai et al., 2019 (estimated by the increase in skatole)	Decreased	Lawler et al., 2021
			Increased	Blasco et al., 2020
Picolinic acid (blood)	Increased	Yau et al., 2014	Increased	Thomas et al., 2020
	Decreased	Nikolaus et al., 2017; Whiley et al., 2019		

### Glutamine

Glutamine is an L-α gluconeogenic and proteogenic amino acid containing five carbons and two amino groups. L-glutamine is considered a conditionally essential amino acid; it is obtained mainly through the diet, as well as it is synthesized *de novo* from glutamate and ammonia in almost all the human cells by the activity of glutamine synthase (E.C.: 6.3.1.2.). In rapidly diving cells, such as enterocytes of the small intestine (Windmueller and Spaeth, 1974), lymphocytes, neutrophils, macrophages, and tumor cells, as well as under catabolic stressed conditions, including severe infections and sepsis, the endogenous synthesis does not meet the energy cell demand and the amount deriving from digested food absorbed through the small intestine becomes vitally important (Cruzat et al., 2018). Glutamine is involved in many cytoplasmatic and mitochondrial pathways, such as (a) the preservation of the reactive oxygen species (ROS) homeostasis, by contributing to the synthesis of the glutathione (Matés et al., 2002); (b) the biosynthesis of hexosamine, nucleotides, asparagine; and (c) the activation of glutaminolysis (Yoo et al., 2020). In the gut, the role of L-glutamine is crucial (Kim and Kim, 2017). Firstly, glutamine exerts an anti-inflammatory activity, preventing the expression of pro-inflammatory cytokines through the inhibition of both nuclear factor *k* light chain-enhancer of activated B cells (NF-*k*B) and signal transducer and activator of transcription (STAT) proteins (Kretzmann et al., 2008). Secondly, L-glutamine enhances tight junction integrity, as demonstrated in animal models (Wang et al., 2016), triggering the mitogen-activated protein kinase (MAPK) function (Basuroy et al., 2006; Perna et al., 2019); in addition, L-glutamine is pivotal for gut cells proliferation (Rhoads et al., 1997). Finally, L-glutamine modulates NO synthetase expression (Hecker et al., 1990; Swierkosz et al., 1990). Thus, it is not surprising that L-glutamine is the most abundant free amino acid in humans. L-glutamine provides energy as a substitute fuel to the tricarboxylic acid (TCA) cycle to produce adenosine triphosphate (ATP; Curi et al., 2005); recently, it was postulated that L-glutamine is the fuel for the immune system, generating the concept of immunometabolism (Wang et al., 2019).

In IBD, L-glutamine plasma levels are decreased (Table 4), especially during the acute exacerbation of CD (Sido et al., 2006; Bjerrum et al., 2010; Ooi et al., 2011; Scoville et al., 2018). Supplementation improves inflammation (Sugihara et al., 2019) and the mucosal barrier integrity in patients with remissive CD (Benjamin et al., 2012b). Glutamine is reduced in the blood of patients with non-severe COVID19 and much more in severe forms (Thomas et al., 2020; Doğan et al., 2021; Meoni et al., 2021; Table 4). In patients with COVID-19, it was observed a 19% reduction in glutamine blood level compared to that before the onset of the disease (Bruzzone et al., 2020). This finding may be related to higher consumption of gluconeogenic amino acids, especially in patients with severe forms, because of the significant scarcity of amino acids (Fanos et al., 2021). A further possible explanation may be the increased conversion of L-glutamine into glutamate, supported by the blood Krebs cycle’s intermediates elevation and by the increase in oxidative stress (Páez-Franco et al., 2021).

**Table 4:** Changes in the concentration of various amino acids in patients with IBD and COVID-19.

Metabolite	Inflammatory bowel disease (IBD)		Coronavirus disease 2019 (COVID-19)	
	Trend	Ref.	Trend	Ref.
L-Glutamine (blood)	Decreased	Sido et al., 2006; Bjerrum et al., 2010; Ooi et al., 2011; Scoville et al., 2018	Decreased	Bruzzone et al., 2020; Thomas et al., 2020; Meoni et al., 2021; Doğan et al., 2021
L-Glutamine (gut mucosa)	Decreased	Ooi et al., 2011		
Histidine (blood)	Decreased	Bjerrum et al., 2010; Ooi et al., 2011; Hisamatsu et al., 2012; Kohashi et al., 2014; Dawiskiba et al., 2014; Bosch et al., 2018; Probert et al., 2018	Decreased	Thomas et al., 2020; Barberis et al., 2020; Bruzzone et al., 2020; Kimhofer et al., 2020; Lawler et al., 2021; Meoni et al., 2021
Histidine (stools)	Decreased	Kolho et al., 2017		
Histidine (stools)	Increased	Kolho et al., 2017		
Phenylalanine (serum)	Increased	Zhang et al., 2013; Dawiskiba et al., 2014	Decreased	Kimhofer et al., 2020; Shi et al., 2021
			Increased	Barberis et al., 2020; Bruzzone et al., 2020
Phenylalanine (urine)	Increased	Alonso et al., 2016		
Phenylalanine (stools)	Increased	Jansson et al., 2009; Kolho et al., 2017; Bosch et al., 2018		
Succinate (blood, urine)	Increased	Ooi et al., 2011; Macias-Ceja et al., 2019	Increased	Barberis et al., 2020; Bruzzone et al., 2020
	Decreased	Schicho et al., 2012; Stephens et al., 2013; Dawiskiba et al., 2014	Decreased	Song et al., 2020
Succinate (gut mucosa)	Increased	Macias-Ceja et al., 2019		
	Decreased	Ooi et al., 2011		
Citrate (blood, urine)	Decreased	Schicho et al., 2012; Stephens et al., 2013; Dawiskiba et al., 2014	Decreased	Páez-Franco et al., 2021
			Increased	Bruzzone et al., 2020
Citrate (gut mucosa)	Decreased	Ooi et al., 2011		

### Histidine

Histidine is an essential amino acid playing a crucial role as a ROS scavenger and anti-inflammatory mediator (Son et al., 2005; Holeček, 2020). The decarboxylation of histidine, catalyzed by histidine decarboxylase, originates histamine, a primary mediator in allergic diseases and a neurotransmitter involved in the control of food intake and sleep biorhythm. Histidine decarboxylase is expressed in bacteria of the large intestine and human muscle, liver, lung, and gastric mucosa (Moro et al., 2020). Blood histidine has been found significantly decreased in patients with IBD (Bjerrum et al., 2010; Ooi et al., 2011; Dawiskiba et al., 2014; Kohashi et al., 2014; Bosch et al., 2018; Probert et al., 2018). An early study found that histidine was significantly decreased in a large cohort (*n* = 387) of IBD patients (Hisamatsu et al., 2012); interestingly, plasma histidine was significantly lower in patients with active disease than in those in remission. Furthermore, a significant inverse correlation was observed between plasma histidine and serum CRP in patients with UC and CD. Authors postulated that the decrease in plasma histidine may reflect chronic inflammation in patients with IBD, suggesting supplementation with histidine as a novel therapy. In a subsequent 1-year follow-up of patients with UC in clinical remission, the same research group found that the decrease in histidine plasma level over time was associated with the increased risk of clinical relapse (Hisamatsu et al., 2015). Previous *in vitro* studies and in animal models suggested that low levels of histidine do not allow the effective suppression of NF-*k*B (Andou et al., 2009; Hasegawa et al., 2011); thus, LPS-induced TNF-α expression cannot be inhibited, resulting in the exacerbation of inflammation. This was confirmed by the amelioration of intestinal inflammation after oral administration of histidine. A recent metabolomics-based study on serum and stool samples in pediatric patients with IBD found controversial results. Compared with healthy controls, fecal histidine was significantly decreased in children with CD and significantly increased in those with UC (Kolho et al., 2017). Moreover, in the group of children with UC, serum histidine inversely correlated with erythrocyte sedimentation rate (ESR). Histidine fecal levels have been positively associated with more extended disease in UC but not in CD pediatric patients (Jagt et al., 2021). A possible explanation may be either the colonic leakage of histidine and other amino acids or malabsorption. However, the overgrowth of *Bacteroides vulgatus* in patients with UC leads to an increased fecal proteolytic and elastase activity (Galipeau et al., 2021). Therefore, it is reasonable to assume that the increased proteolytic and elastase activity might be the main factor promoting the high concentrations of fecal histidine rather than the protein-losing enteropathy. Several studies found L-histidine significantly decreased in the serum/plasma of patients with COVID-19 (Table 4; Barberis et al., 2020; Bruzzone et al., 2020; Thomas et al., 2020); the magnitude of L-histidine decline correlated with the disease severity (Lawler et al., 2021; Meoni et al., 2021). COVID-19 is marked by the activation of gluconeogenesis that is positively correlated with the severity of the disease. In patients with COVID-19, it was observed a 16% reduction in L-histidine blood levels compared to those before the onset of the disease, similarly to glutamine (Bruzzone et al., 2020). In COVID-19, low L-histidine serum levels may be related to the skeletal muscle breakdown (Kimhofer et al., 2020).

### Phenylalanine

Phenylalanine is an aromatic, hydrophobic essential amino acid involved in the biosynthesis of catecholamines (Matthews, 2007). Phenylalanine is hydroxylated to tyrosine by phenylalanine 4-hydroxylase; this reaction primarily occurs in the liver and the kidney. In turn, tyrosine is hydroxylated to L-DOPA (3,4-Dihydroxyphenylalanine) by tyrosine-5 hydrolase, and the enzyme DOPA-decarboxylase converts L-DOPA into Dopamine. A restricted number of gut bacteria, mainly belonging to the phylum *Firmicutes,* such as *Clostridium sporogenes* and *C. botulinum* spp., metabolize aromatic amino acids tryptophan, phenylalanine, and tyrosine to their corresponding propionic acid derivatives, namely, phenylpropionic acid and 4-hydroxyphenylpropionic acid (Elsden et al., 1976). In *C. sporogens*, this metabolic pathway can produce nine metabolites; they accumulate in host serum and exhibit specificity by engaging receptors and altering host biology, especially systemic immunity and gut permeability (Dodd et al., 2017). Although an early metabolomics-based study focused on amino acids did not report any data on phenylalanine level in stool samples from adults with IBD (Marchesi et al., 2007), further studies found a significant increase of phenylalanine in serum (Zhang et al., 2013; Dawiskiba et al., 2014), urine (Alonso et al., 2016), and stools (Jansson et al., 2009; Kolho et al., 2017; Bosch et al., 2018). Interestingly, the magnitude of fecal phenylalanine increase differed between CD and UC, and no correlation was found between the disease activity and fecal phenylalanine concentration (Bosch et al., 2018). Given that the activity of phenylalanine-4-hydroxylase is impaired during immune activation and inflammation (Scholl-Bürgi et al., 2013), it is reasonable to assume that the increase of phenylalanine in patients with IBD may originate from the accumulation of this amino acid. A further contribution to the fecal phenylalanine increase in IBD may be related to the microbial biosynthesis of aromatic amino acids *via* the shikimate pathway (Sprenger, 2006). The condensation of phosphoenolpyruvate with erythrose 4-phosphate, deriving from the glycolysis pathway and the non-oxidative branch of the pentose phosphate pathway, respectively, yields 3-deoxy-d-arabino-heptulosonate-7-phosphate (DAHP), which is converted into chorismic acid and then into aromatic amino acids L-phenylalanine, L-tyrosine, and L-tryptophan. Approximately one-third of gut bacteria expresses all the transcripts coded from the genes of the shikimate pathway, including *A. muciniphila* (Mesnage and Antoniou, 2020); the remaining bacteria species do not exhibit a complete shikimate pathway, having lost either one enzyme (nearly 22%) or five or more enzymes (nearly 74%; Zucko et al., 2010).

In COVID-19, the considerable generation of ROS, due to cytokine activation, induces the irreversible non-enzymatic oxidation of 5, 6, 7, 8-tetrahydrobiopterin (BH4), a cofactor of phenylalanine 4-hydroxylase (PAH), the enzyme catalyzing the conversion of phenylalanine to tyrosine. BH4 shortage induces the loss of PAH activity, with the reduced biosynthesis of tyrosine and the accumulation of phenylalanine. Data on phenylalanine emerging from metabolomic studies in patients with COVID-19 are heterogeneous and discordant: both phenylalanine and tyrosine were decreased, especially in severe and fatal outcomes (Barberis et al., 2020) or increased (Kimhofer et al., 2020; Shi et al., 2021). Interestingly, in non-survivors, phenylalanine serum levels are significantly higher than in survivors (Shi et al., 2021). Moreover, the increase in phenylalanine was associated with the decrease in tyrosine (Bruzzone et al., 2020). The role of phenylalanine in SARS-CoV-2 infection seems to be crucial. In a multicenter study on elderly patients with COVID-19, phenylalanine and tyrosine metabolisms were highly upregulated in 132 deceased patients (median age 74 years) compared with 91 survivors (median age 70 years), suggesting that these amino acids contribute as building blocks for the production of internal SARS-CoV-2 protein and its subsequent assembly into viral particles (Mei et al., 2021).

### Succinate and Citrate

Succinate and citrate are intermediates of the TCA cycle. Succinate is generated within mitochondria *via* the TCA cycle from succinyl-CoA; then, succinate is oxidized to fumarate by succinate dehydrogenase. Succinate is also a product of the glyoxylate cycle, a pathway active in many bacteria, plants, and fungi. Overall, succinate accumulation is a metabolic signature of anoxia, asphyxia, and ischemia; the dysregulation of succinate metabolism can lead to pathological conditions, such as malignant transformation, inflammation, and tissue injury. Metabolomics-based studies carried out in serum, plasma, and urine of patients with IBD found that succinate and citrate significantly decreased in CD and UC compared to non-IBD individuals (Schicho et al., 2012; Stephens et al., 2013; Dawiskiba et al., 2014). Succinate and citrate are strongly involved in energy metabolism, and their depletion confirms the increased demand and the rapid utilization of cellular energy in IBD. In this context, citrate depletion may be associated with the increase in fatty acids biosynthesis. Indeed, citrate is essential for carrying acetyl-CoA from mitochondria to the cytosol. The significant increase in circulating triglycerides, observed in several studies on IBD, can be considered further evidence (Levy et al., 2000; Sappati Biyyani et al., 2010; Tefas et al., 2020). Beyond the role of succinate as an energy supplier, this amino acid is an inflammation mediator; it selectively binds to and activates the succinate receptor-1 (SUCNR1), which promotes pro-inflammatory signaling pathways (Mills and O'Neill, 2014). In patients with CD, hypoxia, inflammation, and necrosis promote the accumulation of succinate in gut inflamed areas, with a further activation and infiltration of macrophages and fibroblasts, the overexpression of pro-inflammatory cytokines, and ultimately the acceleration of fibrosis (Macias-Ceja et al., 2019).

Data on succinate emerging from metabolomics-based studies in COVID-19 are controversial (Table 4); the concentration of this amino acid has been found increased (Barberis et al., 2020; Bruzzone et al., 2020) decreased in moderate and severe disease (Song et al., 2020), or unchanged (Thomas et al., 2020). Differences between patients normo-oxygenate and patients undergoing intense respiratory treatment may affect results obtained in different studies. In the paper of Bruzzone, the increase in succinate and citrate (156 and 12%, respectively) has been associated with central carbon metabolism dysfunction (Bruzzone et al., 2020). In severe COVID-19, the association of citrate decrease with succinate increase could be related to mitochondrial dysfunction due to hypoxia (Páez-Franco et al., 2021). Hypoxia inhibits oxidative phosphorylation, and thus energy is supplied by the anaerobic glycolysis, which is activated by the accumulation of the hypoxia-inducible factor 1α (HIF 1α; Majmundar et al., 2010). As a result, the TCA is blocked, with the consequent accumulation of succinate, depletion of citrate, and increased demand for glucose. In turn, the latter induces the decreased availability of gluconeogenic amino acids and oxaloacetate, being utilized as substrates for gluconeogenesis. Oxaloacetate is converted into glucose, whereas mitochondrial acetyl-CoA oxidation is drastically reduced, and acetyl-CoA is redirected to the synthesis of ketone bodies.

### Ketone Bodies

Ketone bodies are small lipid-derived molecules, namely, 3-β-hydroxybutyrate, acetone, and acetoacetate (Laffel, 1999). During fasting or prolonged exercise, the liver converts fatty acids mobilized from adipocytes into ketone bodies; then, they enter circulation, serving as an energy source. Ketone bodies are crucial regulators of metabolic health and longevity; in fact, they are neuroprotective and cytoprotective (Yang et al., 2019), having the ability to inhibit histone deacetylase activity and thereby epigenetic gene regulation (Newman and Verdin, 2014). On the one hand, ketone bodies were increased in the serum of patients with IBD (Table 5), and this finding was correlated to the increased energy demand (Zhang et al., 2013; Dawiskiba et al., 2014; Kohashi et al., 2014; Keshteli et al., 2017). On the other hand, ketone bodies may play a strategic role in IBD when supplemented with diet, thanks to their capacity to protect from toxic effects of chronic inflammation. An experimental study demonstrated that 3-β-hydroxybutyrate suppresses the activation of the NLRP3 inflammasome in response to urate crystals, ATP, and lipotoxic fatty acids (Youm et al., 2015). Then, in a mouse model of NLRP3-mediated diseases, authors observed that 3-β-hydroxybutyrate attenuates caspase-1 activation and the release of pro-inflammatory cytokines IL-1β and IL-18 from macrophages, reducing in definitive the severity of NLRP3-mediated chronic inflammatory diseases. The same effect was obtained by applying a ketogenic diet or supplementing 3-β-hydroxybutyrate (Youm et al., 2015). The accumulation of ketone bodies following a ketogenic diet strongly impacts gut microbiota composition; *in vivo* and *in vitro* experiments demonstrated that ketone bodies selectively inhibit the growth of several *Bifidobacterial* spp., with downstream consequences for gut immune cells, especially Th17, and induce the decrease in the relative abundance of *Actinobacteria* (Ang et al., 2020). In an animal model of inflammatory colitis, it was observed that the ketogenic diet alters gut microbiota and serum metabolome, alleviating colitis (Kong et al., 2021). In particular, *Akkermansia*, *Roseburia*, and *Ruminococcaceae* genera were enriched. After colitis induction, the ketogenic diet protected intestinal barrier function and reduced the presence of RORgt^+^CD3^−^ group 3 innate lymphoid cells and related inflammatory cytokines (IL-17α, IL-18, IL-22, and Ccl4). As a result, the ketogenic diet in patients with IBD may substantially contribute to control inflammation and shape gut microbiota.

**Table 5:** Changes in lipid concentration in IBD and in COVID-19.

Metabolite	Inflammatory bowel disease (IBD)		Coronavirus disease 2019 (COVID-19)	
	Trend	Ref.	Trend	Ref.
3-β-hydroxybutyrate	Increased	Zhang et al., 2013; Dawiskiba et al., 2014; Kohashi et al., 2014; Keshteli et al., 2017	Increased	Barberis et al., 2020; Bruzzone et al., 2020; San Juan et al., 2020; Páez-Franco et al., 2021; Meoni et al., 2021
Acetone	Increased	Keshteli et al., 2017	Increased	Bruzzone et al., 2020; San Juan et al., 2020
Acetoacetate	Increased	Dawiskiba et al., 2014; Keshteli et al., 2017	Increased	Bruzzone et al., 2020; San Juan et al., 2020
Glycerophospholipids	Decreased	Bjerrum et al., 2010, 2017; Fan et al., 2015; Kolho et al., 2017; Scoville et al., 2018; Tefas et al., 2019, 2020	Decreased	Barberis et al., 2020; Wu et al., 2020; Song et al., 2020; Shen et al., 2020; Schwarz et al., 2021
Lysophospholipids	Decreased		Increased	Barberis et al., 2020; Song et al., 2020; Schwarz et al., 2021
Sphingolipids	Decreased	Fan et al., 2015; Kolho et al., 2017	Decreased	Barberis et al., 2020; Shen et al., 2020; Schwarz et al., 2021
			Increased	Song et al., 2020
Arachidonic acid (blood)	Decreased	Esteve-Comas et al., 1992; Scoville et al., 2018; Lai et al., 2019; Manfredi et al., 2019	Increased	Barberis et al., 2020; Schwarz et al., 2021; Thomas et al., 2020
Arachidonic acid (stools)	Increased	Jansson et al., 2009		

Ketone bodies accumulate in the serum of patients with COVID-19, mimicking diabetic ketoacidosis (Li et al., 2020). Further studies confirmed the elevation of circulating ketone bodies in COVID-19 (Table 5), suggesting their role as an alternative energy source during SARS-CoV-2 replication (Barberis et al., 2020; Bruzzone et al., 2020; San Juan et al., 2020; Meoni et al., 2021; Páez-Franco et al., 2021). The increase in 3-β-hydroxybutyrate could interfere with viral replication by upregulating the expression of antioxidant genes, the cytoplasmic NADPH, and directly scavenging free radicals (Stubbs et al., 2020). In addition, 3-β-hydroxybutyrate may induce the closing of mitochondrial permeability transition pore, apoptosis, and the inhibition of glycolysis. It was postulated that ketone bodies inactivate the extracellular virions (Shaheen, 2021). Ketone bodies have carbonyl groups reacting with the ε-amino group of lysine to form a Schiff base. Given that SARS-CoV-2 spike protein contains approximately 4.5% lysine residues, almost equally distributed between the two subunits, the reaction between the ketone body, mainly acetoacetate, and the lysine residues of the spike protein forms Schiff bases, altering the conformation of the spike protein. This change promotes the separation of the spike protein from the virion, either by the separation between S1 and S2 subunits or promoting its bending. As a result, acetoacetate induces protein conformational changes by altering the secondary structure, namely, reducing the α-helix content (Bohlooli et al., 2016). The sum of these researches has raised the question of whether or not it may be effective to induce ketosis both in asymptomatic individuals infected with SARS-CoV-2 and in patients with COVID-19 (Bradshaw et al., 2020).

### Phospholipids

Glycerophospholipids, commonly termed phospholipids, their by-product lysophospholipids, and sphingolipids are key components of the cellular membrane; remarkably, phospholipids are involved in the metabolism of cell signaling. Phospholipids are essential for the biosynthesis of lipoproteins. As reported in Table 5, in patients with IBD, circulating phospholipids were found decreased in various studies (Bjerrum et al., 2010, 2017; Fan et al., 2015; Kolho et al., 2017; Scoville et al., 2018; Tefas et al., 2019, 2020). Factors, such as the compromised integrity of the intestinal mucosa, TNF-α, NF-*k*B, mitogen-activated protein kinase (MAPK) pathway, and peroxisome proliferator-activated receptor (PPAR) signaling, seem to be closely implicated in phospholipids depletion. Glycerophosphocholine was significantly decreased in two subsequent studies (Bjerrum et al., 2010, 2017); more recently, in patients with extensive UC and colonic CD, tetracosanoic acid, phosphatidylcholine, (PC) lysophosphatidylcholine (LPC), sphingomyelin (SM), and diacylglycerol were found decreased compared with healthy controls; interestingly, saturated LPC (18:2) was found decreased whereas polyunsaturated LPC (20:4) and LPC (22:6) increased (Tefas et al., 2019, 2020). It is likely that the anti-inflammatory polyunsaturated LPC effectively antagonized the pro-inflammatory activity of saturated LPC. Two studies found sphingolipids significantly reduced in patients with IBD (Fan et al., 2015; Kolho et al., 2017). The significant depletion in sphingolipids may be the result of the increased activity of sphingomyelinases, which are activated in IBD by the combined action of TNF-α, NF-*k*B, and IFN-γ (Schütze et al., 1992).

Phospholipids metabolism is strongly influenced by COVID-19 and by the severity of the disease (Mussap and Fanos, 2021); data emerging from the literature suggest that SARS-CoV-2 infection promotes the downregulation of most phospholipids and sphingolipids (Table 5), while various lysophospholipids are either overexpressed or unchanged (Barberis et al., 2020; Shen et al., 2020; Song et al., 2020; Wu et al., 2020; Schwarz et al., 2021). The downregulation of phospholipids may originate from the liver impairment in patients with severe COVID-19, whereas lysophospholipids upregulation is the result of the increased activity of phospholipase A_2_. Low-risk infected patients are well discriminated from non-infected individuals by high levels of phosphatidylcholine (PC38:8), phosphatidylethanolamine (PE38:4), and phosphatidylglycerol (PG20:5). In patients with COVID-19, the predominance of inflammation over the macrophage-driven anti-inflammatory response leads to the underexpression of sphingosine 1-phosphate (Shen et al., 2020; Song et al., 2020). The considerable number of lipids belonging to any lipid class gives a limited value to the definition of upregulation and downregulation of phospholipids, lysophospholipids, and sphingolipids in COVID-19; recently, a targeted lipidomic analysis measured a considerable number of phospholipids (*n* = 90), lysophospholipids (*n* = 14), and sphingolipids (*n* = 15), discovering that certain lipids decrease and other increase within the same lipid class (Caterino et al., 2021).

### Arachidonic Acid and Phospholipases

Arachidonic acid is a 20-carbon chain belonging to the ω-6 (n-6) polyunsaturated fatty acids (PUFAs); it is obtained from food and then incorporated in phospholipids (Tallima and El Ridi, 2017). Arachidonic acid is the primary precursor for the biosynthesis of eicosanoids, a complex family of lipid signaling mediators including but not limited to leukotrienes, prostaglandins, thromboxane, and prostacyclin A_2_ (Calder, 2020). By the cleavage of arachidonic acid from membrane phospholipids, phospholipases A_2_-IID, -IIF, -III, and -X initiate the arachidonic acid cascade and eicosanoid production (Murakami et al., 2020). Eicosanoids are generated by three main pathways, namely, cyclooxygenases, lipoxygenases, and cytochrome P-450 epoxygenase pathways (Dennis and Norris, 2015). Beyond their crucial role in a broad range of physiological processes, eicosanoids are involved in the pathogenesis of IBD (Alhouayek et al., 2021). During the acute phase of the disease, their concentration significantly increases within the inflamed intestinal mucosa (Wallace, 2019). In patients with IBD, arachidonic acid was increased in feces (Jansson et al., 2009) and decreased in the blood (Table 5); these variations were often associated with the exacerbation of the disease activity (Esteve-Comas et al., 1992; Scoville et al., 2018; Lai et al., 2019; Manfredi et al., 2019; Gallagher et al., 2021). Based on current knowledge, the decrease of circulating arachidonic acid in IBD might be related to the increased synthesis of eicosanoids in the gut (Shores et al., 2011). SARS-CoV-2 infection induces the overexpression of phospholipase A_2_, especially in macrophages, T, and B cells; as a consequence, the biosynthesis of arachidonic acid is upregulated in patients with COVID-19 (Table 5). The arachidonic acid upregulation positively correlates with the IL-6 concentration and the disease’s severity (Barberis et al., 2020; Thomas et al., 2020; Schwarz et al., 2021). In patients with COVID-19 and severe liver injury, however, arachidonic acid may be downregulated (Shen et al., 2020). Arachidonic acid is a potent antiviral PUFA that can inactivate the enveloped viruses, including SARS-CoV-2 (Hoxha, 2020); human cells infected by HCoV-229E and MERS-CoV are inhibited by arachidonic acid (Yan et al., 2019). The increased activity of the cytosolic phospholipase A_2_α (cPLA_2_α) in cells infected by SARS-CoV-2 generates the overproduction of lysophospholipids that are essential for the viral replication (Casari et al., 2021); the pharmacological inhibition of cPLA_2_α in human Huh-7 cells infected with coronavirus 229E drastically reduces the viral RNA synthesis, blocking an early step in the replication cycle (Müller et al., 2018). This finding opens new perspectives on the effective treatment of SARS-CoV-2 infection. A recent study performed a targeted lipidomic analysis of bronchoalveolar lavages fluids (BALs) from patients with severe COVID-19 (Archambault et al., 2021). The most relevant finding was the significant increase in several bioactive lipids, such as thromboxane, leukotrienes, and 15-lipoxygenase metabolites derived from linoleic acid, linolenic acid, and dihomo-γ-linolenic acid (Table 5). During the early stage of inflammation, the enzymatic oxygenation of essential fatty acids, including arachidonic acid, eicosapentaenoic acid, docosapentaenoic acid, and docosahexaenoic acid, generates a class of bioactive lipids, the so-called specialized pro-resolving mediators (SPMs; Serhan et al., 2000). This class includes lipoxins, resolvins, maresins, and protectins (Basil and Levy, 2016). Interestingly, BALs from patients with severe COVID-19 were also marked by the increase in SPMs. Increased levels of pro-inflammatory bioactive lipids and anti-inflammatory SPMs have also been reported in serum samples collected from hospital inpatients with a confirmed diagnosis of COVID-19 (Turnbull et al., 2022).

## Conclusive and Prospective Remarks

IBD and COVID-19 are characterized by gut dysbiosis associated with impaired gut barrier function and immune-mediated chronic inflammation. However, COVID-19 can rapidly evolve to multisystemic organ damage due to a dysregulated, fulminant inflammatory response, the so-called “cytokine storm” (Jain, 2020; Hu et al., 2021). The evident imbalance between the number of studies on gut microbiota in IBD and that in SARS-CoV-2 does not exclude a reliable data analysis; we noticed more similarities than differences in gut microbial alterations between IBD and COVID-19 (Table 1). The depletion in *F. prausnitzii*, *E. rectale*, *R, bromii*, *Lachnospiraceae*, *C. leptum* (cluster IV), and the overgrowth of *Enterococcus*, *E. coli*, *Shigella*, *P. mirabilis*, *Fusobacterium*, *Veillonellaceae* are common patterns of dysbiosis, playing a key role in the severity and clinical outcome of IBD and COVID-19. An intriguing dissimilarity between IBD and COVID-19 is the abundance of *A. muciniphila* (Table 1), a Gram-negative bacterium belonging to the *Verrucomicrobia* phylum. *A. muciniphila* colonizes the mucus layer close to gut epithelial cells and is able to degrade mucin sugars and the protein backbone by specific enzymes, such as sialidases and fucosidases, providing carbon and nitrogen for bacteria unable to produce these enzymes (van Passel et al., 2011). Mucus degradation by *A. muciniphila* generates SCFAs, which are strongly involved in promoting host metabolic health. Therefore, *A. muciniphila* has several beneficial effects on host health by reducing inflammation, stimulating mucin biosynthesis and mucus thickness, preserving the integrity of the mucosal barrier, increasing the expression of tight junction proteins (e.g., occluding), and modulating the intestinal adaptative immune response (Ottman et al., 2017; Ansaldo et al., 2019; Ashrafian et al., 2019; Liu et al., 2021). The depletion of *A. muciniphila* in IBD (Table 1), reported by several studies, confirms the well-known inverse relationship between this bacterium and IBD (Rajilić-Stojanović et al., 2013); on the other hand, the enrichment in *A. muciniphila* in patients with COVID-19 has been associated with that of opportunistic pathogens, such as *Enterococcus*, *Staphylococcus*, *Serratia*, *Collinsella*, *Actinomyces*, and many others (Gaibani et al., 2021). Concerns emerge about results on the probiotic strains *Lactobacilli* and *Bifidobacteria*, especially in patients with COVID-19 (Table 1). These strains are Gram-positive, non-spore-forming, lactic acid producer bacteria with the antiviral activity performed by various mechanisms, including the synthesis of antiviral inhibitory metabolites, the upregulation of the protective immune responses, and by competing for nutrients and colonization sites with the virus and, more extensively, with pathogens (Kesika et al., 2021). Five studies reported *Lactobacillus* enrichment in patients with COVID-19 (Table 1), confuting the general notion that gut dysbiosis due to severe infections promotes the depletion of these strains (Harper et al., 2021). Very few data support the depletion of *Lactobacillus* and *Bifidobacterium* in patients with COVID-19. Two articles cited in the PubMed library, written in Chinese with the same English abstract and the same digital object identifier (doi), described gut dysbiosis in “some patients” with COVID-19 marked by the decrease in *Lactobacillus* and *Bifidobacterium* abundance (Xu et al., 2020a,b). Clearly, these data are unreliable. Therefore, further studies are required to elucidate the significance of *Lactobacillus* and *Bifidobacterium* enrichment in COVID-19.

Metabolomics reveals considerable similarities in the tryptophan metabolism between IBD and COVID-19 (Table 3). In IBD, the increase in quinolinic acid is associated with the decrease in picolinic acid, a non-selective metal ion chelating agent with antimicrobial, antiviral, and antifungal activity formed by a non-enzymic cyclization of aminomuconic acid semialdehyde. In COVID-19, the increase in quinolinic acid is associated with the increase of kynurenic acid; the biochemical mechanism underlying this unusual association should be clarified. In fact, quinolinic acid and kynurenic acid are closely related to each other by an inverse relationship that becomes imbalanced in various diseases. In COVID-19, the increase in picolinic acid reflects the activation of the enzymatic conversion of 2-amino-3-carboxymuconate-6-semialdehyde (ACMS) to 2-aminomuconic-6-semialdehyde. In turn, the latter undergoes either non-enzymatic cyclization to form picolinic acid or enzymatic transformation to 2-aminomuconic acid, yielding acetyl-CoA (Badawy, 2017). It is unclear why picolinic acid is increased in COVID-19, taking into account the alteration of brain functions during the disease (Chou et al., 2021; Marshall, 2021). It is reasonable to assume that the limited number of metabolic differences between IBD and COVID-19 (Tables 4, 5), for example, blood arachidonic acid, originates from the acute systemic damage and impairment of organs, tissues, and biological systems (e.g., coagulation) in COVID-19, while IBD remains a chronic disease localized in the gastrointestinal tract with a broad spectrum of extraintestinal symptoms and comorbidities (Argollo et al., 2019). Emerging evidence indicates the role of the microbiome in modulating the immune response to vaccination and the role of metabolic profile in predicting vaccination outcome (Hagan et al., 2019; Alexander et al., 2021). Therefore, microbiomics and metabolomics may be considered powerful tools for the early identification and monitoring of individuals at risk of adverse events, for example, fragile individuals or patients with severe chronic diseases. Since the COVID-19 pandemic is far from over, SARS-CoV-2 vaccination is the mainstay for preventing the COVID-19 spread. As a result, there is the need to extend SARS-CoV-2 vaccination to any adult or young subject, even if affected by pre-existing chronic diseases, such as IBD (Alexander et al., 2021). Therefore, deciphering the individual metabolic and microbial fingerprint in IBD is crucial to define an effective strategy for the safe administration of the vaccine (e.g., to discover any metabolic/microbial alteration induced by recent steroid or rituximab therapy), to manage vaccinated individuals, and to avoid any possible adverse effect in individuals at risk (Ferretti et al., 2021). On the other hand, deciphering the individual metabolic and microbial fingerprint in patients with IBD with COVID-19 may considerably improve the therapeutic approach, preventing the risk of adverse outcomes.

## Author Contributions

GC and FB contributed to the conception and design of the study. VF conceptualized and performed the modeling. GC wrote the first draft of the manuscript. MM supervised the manuscript and wrote the final version of the paper. All authors contributed to the article and approved the submitted version.

## Conflict of Interest

The authors declare that the research was conducted in the absence of any commercial or financial relationships that could be construed as a potential conflict of interest.

## Publisher’s Note

All claims expressed in this article are solely those of the authors and do not necessarily represent those of their affiliated organizations, or those of the publisher, the editors and the reviewers. Any product that may be evaluated in this article, or claim that may be made by its manufacturer, is not guaranteed or endorsed by the publisher.
